# Hepatitis C Related Chronic Liver Cirrhosis: Feasibility of Texture Analysis of MR Images for Classification of Fibrosis Stage and Necroinflammatory Activity Grade

**DOI:** 10.1371/journal.pone.0118297

**Published:** 2015-03-05

**Authors:** Zhuo Wu, Osamu Matsui, Azusa Kitao, Kazuto Kozaka, Wataru Koda, Satoshi Kobayashi, Yasuji Ryu, Tetsuya Minami, Junichiro Sanada, Toshifumi Gabata

**Affiliations:** 1 Department of Radiology, Kanazawa University Graduate School of Medical Science, 13–1 Takaramachi, Kanazawa 920–8640, Japan; 2 Department of Radiology, Sun Yat-Sen Memorial Hospital, Sun Yat-Sen University, 107 Yan Jiang Xi Road, Guangzhou 510120, Guangdong, China; National Taiwan University Hospital, TAIWAN

## Abstract

**Purpose:**

To assess the feasibility of texture analysis for classifying fibrosis stage and necroinflammatory activity grade in patients with chronic hepatitis C on T2-weighted (T2W), T1-weighted (T1W) and Gd-EOB-DTPA-enhanced hepatocyte-phase (EOB-HP) imaging.

**Materials and methods:**

From April 2008 to June 2012, MR images from 123 patients with pathologically proven chronic hepatitis C were retrospectively analyzed. Texture parameters derived from histogram, gradient, run-length matrix, co-occurrence matrix, autoregressive model and wavelet transform methods were estimated with imaging software. Fisher, probability of classification error and average correlation, and mutual information coefficients were used to extract subsets of optimized texture features. Linear discriminant analysis in combination with 1-nearest neighbor classifier (LDA/1-NN) was used for lesion classification. In compliance with the software requirement, classification was performed based on datasets from all patients, the patient group with necroinflammatory activity grade 1, and that with fibrosis stage 4, respectively.

**Results:**

Based on all patient dataset, LDA/1-NN produced misclassification rates of 28.46%, 35.77% and 20.33% for fibrosis staging and 34.15%, 25.20% and 28.46% for necroinflammatory activity grading in T2W, T1W and EOB-HP images. In the patient group with necroinflammatory activity grade 1, LDA/1-NN yielded misclassification rates of 5.00%, 0% and 12.50% for fibrosis staging in T2W, T1W and EOB-HP images respectively. In the patient group with fibrosis stage 4, LDA/1-NN yielded misclassification rates of 5.88%, 12.94% and 11.76% for necroinflammatory activity grading in T2W, T1W and EOB-HP images respectively.

**Conclusion:**

Texture quantitative parameters of MR images facilitate classification of the fibrosis stage as well as necroinflammatory activity grade in chronic hepatitis C, especially after categorizing the input dataset according to the activity or fibrosis degree in order to remove the interference between the fibrosis stage and necroinflammatory activity grade on texture features.

## Introduction

Hepatitis C related liver cirrhosis remains an important cause of morbidity and mortality [1], and predisposes to hepatocellular carcinoma [2]. Liver biopsy is routinely used to diagnose and stage the severity of hepatitis in patients with chronic hepatitis C [3], but has the drawbacks of invasiveness, sampling error and inherent risks [4]. A noninvasive accurate method to evaluate liver cirrhosis would reduce the risks and costs for extra examinations and facilitate prompt assessment in clinical practice for hepatitis C related liver cirrhosis.

Texture analysis is a new useful post-processing method for radiological images that provides more than the perceivable texture feature parameters. In addition, all parameters of texture features are expressed as numerical values that quantitatively evaluate images. These texture features include histogram, gradient, gray-level co-occurrence matrix, run-length matrix, autoregressive model and wavelet transform parameters [5]. Histogram, co-occurrence matrix, run-length matrix, and gradient come from statistical approaches; autoregressive model is yielded by model-based methods; wavelet is based on transform method [5]. Simply, these texture parameters reflect the extent of heterogeneity, granularity, randomness and so on. Thus, they represent characteristics that may be associated with the histopathological changes and contribute to the differential diagnosis or development grade assessment. Until now, texture analysis has provided a wide range of applications to detect the progression of disease or evaluate the response to treatment [6–8].

The pathogenesis of liver cirrhosis in chronic hepatitis C is progressive fibrosis, namely fibrous scarring that may lead to architectural distortion [9]. In MR images, cirrhotic liver parenchyma is characterized by a heterogeneous intensity that is determined by the underlying pathological features involving regenerating nodules and fibrotic scars, and these alterations are exacerbated with progression of the cirrhosis. Contrast enhancement may emphasize the heterogeneous liver background to stage the grade of hepatic cirrhosis [10], especially gadolinium ethoxybenzyl diethylenetriaminepentaacetic acid (Gd-EOB-DTPA) because of its liver-specific character and excellent enhancement of liver parenchyma on the hepatobiliary phase of Gd-EOB-DTPA enhanced MR imaging (EOB-HP) [11].

Since texture analysis has hitherto succeeded in separating cirrhotic patients and healthy volunteers [12], the impact of texture features measured on MR images is expected to further define the diagnostic performance of discrimination of various fibrotic stages and activity grades. The aim of this study was to investigate whether the texture features extracted from T1W, T2W and EOB-HP images can differentiate fibrosis stages or necroinflammatory activity grades in patients with chronic hepatitis C.

## Materials and Methods

### Patients

The approval of this retrospective analysis of the daily clinical data was waived by Kanazawa University Graduate School of Medical Science institutional review board. Written informed consent for the use of daily clinical data, including imaging materials, was obtained from all patients at the time of their admission. Patient records/information was not anonymized and de-identified prior to analysis, because we should check the clinical data of these patients during analysis. We enrolled 158 patients with pathology-proven chronic hepatitis C who underwent Gd-EOB-DTPA-enhanced MR examination from April 2008 to June 2012. Inclusion criteria included the interval between MR examination and biopsy or resection (within 90 days) and the absence of any evident artifacts in the T1W, T2W and EOB-HP images. 35 patients were excluded because the interval between MR examination and biopsy or resection was over 90 days in 28 patients and evident imaging artifacts in T2W images in 7 patients, leaving 123 patients (75 men, mean age 68.4 years, range 52~84 years; 48 women; mean age 70.2 years, range 50~86 years) for analysis. Child-Pugh classification of the patients was as follows: Child-Pugh A (n = 86), Child-Pugh B (n = 33) and Child-Pugh C (n = 4).

The histopathology (liver biopsy, n = 104; resection, n = 19) was interpreted by one of two board-certified pathologists, who were blinded to the imaging results, as part of routine clinical practice. The necroinflammatory activity grades were classified as follows: A1, mild activity; A2, moderate activity; and A3, severe activity. Criteria for the fibrosis stages were as follows: F1, fibrous portal expansion; F2, bridging fibrosis; F3, bridging fibrosis with architectural distortion; and F4, cirrhosis [13].

### MRI examinations

T2-weighted images were obtained with frequency-selective fat saturation sequence with the following parameters: TR/TE = 15000/89 ms, flip angle 90°, field of view 40×40 cm, matrix 512×512, slice thickness 4.0 mm. T1-weighted images were obtained with fat-suppressed 3D SPGR T1-weighted sequence with the following parameters: LAVA-XV, GEM, TR/TE = 3.2–4.0/1.6 ms, flip angle 6–15°, field of view 42×42 cm, matrix 192×320 (512 interpolated), slice thickness 4.2 mm. A dose of 0.1 ml/kg of Primovist (0.25 mmol/ml of gadoxetic acid, Bayer Schering Pharma, Berlin) was intravenously injected at a flow rate of 1.0 ml/sec, followed by a 30-ml saline flush. The hepatobiliary phase was obtained 20 min after the injection.

### Texture analysis and classification

**Fig 1:**
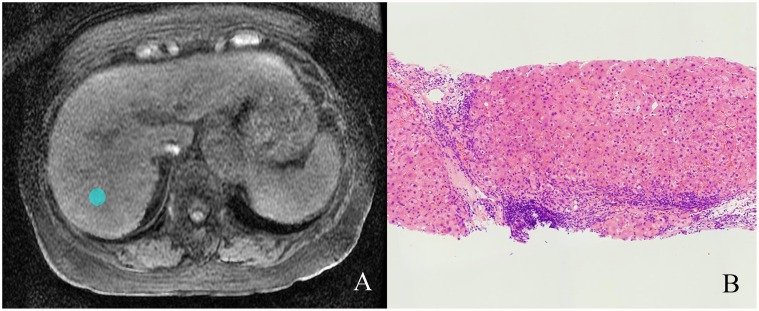
Illustration of region of interest (ROI). **(A)** One 20-pixel diameter ROI was placed in the axial T1W images (3.6/1.6; flip angle, 12°) at the level of the porta hepatis in a 76-year-old woman with chronic hepatitis C. **(B)** Liver biopsy specimen showed stage 4 hepatic fibrosis and grade 2 necroinflammatory activity. (Hematoxylin-eosin stain; original magnification, ×50.)

For each sequence (T1W, T2W and EOB-HP), one slice at the level of the porta hepatis was selected and then loaded in bitmap format in the texture analysis software, MaZda 4.6 (URL: http://www.eletel.p.lodz.pl/programy/mazda/) [14–16]. A circular region of interest (ROI) was placed in the right lobe of the liver parenchyma devoid of large vessels, hepatic lesions, and artifacts by an experienced radiologist with 12 years of clinical experience (Fig. 1). To keep the ROI consistent, each 20-pixel diameter ROI was loaded from a file containing a standard-size ROI box. Altogether 279 descriptors were produced automatically and derived from the gray-level histogram (n = 9, the basic characters reflecting image uniformity) [15], gradient (n = 5, a direction change in grey level intensity representing the image intensity distribution) [15], the run-length matrix (n = 20, calculated from different run angles in four directions including horizontal, vertical, 45°and 135°, and indicating image coarseness) [17–20], the co-occurrence matrix (n = 220, computed from intensities of pairs of pixels and describing the homogeneity) [15,21–22], the autoregressive model (n = 5, the coefficients of neighboring pixels reflecting the coarse-to-fine stratification) [5, 18, 19], and the wavelet transform (n = 20, the spatial frequencies at multiple scales identifying coarseness) [23–25]. The categories of the texture parameters are listed in Table 1 in detail [26].

**Table 1:** Texture feature list in MaZda software.

Methods	Texture features
Histogram	Mean (histogram’s mean); Variance (histogram’s variance); Skewness (histogram’s skewness); Kurtosis (histogram’s kurtosis); Perc.01% (1% percentile); Perc.10% (10% percentile); Perc.50% (50% percentile); Perc.90% (90% percentile); Perc.99% (99% percentile)
Gradient	GrMean (absolute gradient mean); GrVariance (absolute gradient variance); GrSkewness (absolute gradient skewness); GrKurtosis (absolute gradient kurtosis); GrNonZeros (percentage of pixels with nonzero gradient)
Run-length matrix	RLNonUni (run length nonuniformity); GLevNonU (grey level nonuniformity); LngREmph (long run emphasis); ShrtREmp (short run emphasis); Fraction (fraction of image in runs)
Co-occurrence matrix	AngScMom (angular second moment); Contrast (contrast); Correlat (correlation); SumOfSqs (sum of squares); InvDfMom (inverse difference moment); SumAverg (sum average); SumVarnc (sum variance); SumEntrp (sum entropy); Entropy (entropy); DifVarnc (difference variance); DifEntrp (difference entropy). Features are computed for 5-pixel distance (1, 2, 3, 4, 5) and for 4 various directions (horizontal, 45 degrees, vertical, 135 degrees)
Autogressive model	Teta1 (parameter θ1); Teta2 (parameter θ2); Teta3 (parameter θ3); Teta4 (parameter θ4); Sigma (parameter σ)
Wavelet transform	WavEn (wavelet energy) feature is computed at 5 scales within four frequency bands: low-pass filtering in both directions (LL) assessed the lowest frequencies, low-pass filtering followed by high-pass filtering (LH) assessed horizontal edges, high-pass filtering followed by low-pass filtering (HL) assessed vertical edges; high-pass filtering in both directions (HH) assessed diagonal details.

Gray-level intensity normalization was run in the texture analysis application separately for each ROI, with method-limiting image intensities in the range μ-3σ, μ+3σ, where μ is the mean gray-level value and σ the standard deviation, to minimize the influence of contrast and brightness variation, which might otherwise obscure the true image texture [26].

Following the texture parameter calculation, feature reduction was performed. Based on a pooled texture dataset of all patients, the 30 most discriminating texture features were selected automatically from 279 parameters using MaZda program for distinguishing fibrosis stages or necroinflammatory activity grades based on the combination of three methods (Fisher coefficients [Fisher]; classification error probability [POE] combined with average correlation coefficients [ACC]; mutual information coefficients [MI]) as Fisher+POE+ACC+MI separately for T2W, T1W and EOB-HP images [27]. Then the patients were divided into 12 subgroups in terms of both fibrosis stages (F1~4) and necroinflammatory activity grades (A1~3). Since the software requirement is that the sample size for each subgroup must exceed one, but the sample size for F1A2, F1A3, F2A3 and F3A3 subgroup was less than or equal to one, only A1 and F4 groups met this condition. Further texture feature reduction was performed for fibrosis staging of A1 group and necroinflammatory activity grading of F4 group. With the same selection method Fisher+POE+ACC+MI as before, the 30 most discriminating texture features were selected in texture datasets of A1 group and F4 group, separately. A total of 12 groups of 30 parameters were obtained.

B11 application (version 3.4) of MaZda software package was used for texture data analysis and classification. Analyses were performed for a combination of 30 parameters selected automatically with Fisher+POE+ACC+MI methods. Linear discriminant analysis (LDA) was run for these chosen texture feature groups, followed by the 1-nearest neighbor (1-NN) classification used for selecting the most discriminating features (MDF). LDA is a supervised feature transformation method to produce new feature vectors, the so-called MDFs, which are optimized for both maximum between-class scatter and minimum within-class scatter [28]. The classification results of LDA were represented graphically as the relationship between MDFs. The number of MDF axes is less than the output classes by one. When 1-NN classifier is used, these subsets are optimal in the sense that they provide minimal classification error [26]. The classification procedures were run by B11 automatically.

### Statistical analysis

For different fibrosis stages or necroinflammatory activity grades, patient age, body mass index (BMI), hepatitis C virus (HCV) RNA titer, alanine transaminase (ALT), aspartate aminotransferase (AST), glucose, total cholesterol, triglyceride, high density lipoprotein (HDL) and low density lipoprotein (LDL) were compared by one-way analysis of variance (ANOVA) followed by Tukey post hoc test; patient sex ratio and HCV serogroup ratio were compared by the Chi-square test or Fisher exact test, with Bonferroni correction.

## Results

The stages of fibrosis and grades of necroinflammatory activity observed among the 123 patients on histopathologic analysis are summarized in Table 2. Patient demographic variables according to fibrosis stage and necroinflammatory activity grade are summarized in Table 3. No significant differences were found in age, BMI, HCV RNA titer, ALT, AST, glucose, total cholesterol, triglyceride, HDL, LDL, HCV serogroup ratio or sex ratio among them.

**Table 2:** Distribution of Various Stages of Fibrosis and Grades of Necroinflammatory Activity.

		Necroinflammatory activity grade			Total
		1	2	3	
Fibrosis stage	1	3	1	0	4
	2	10	3	0	13
	3	12	8	1	21
	4	15	68	2	85
Total		40	80	3	123

Data indicate number of patients.

**Table 3:** Demographic variables classified by fibrosis stage and necroinflammatory activity grade.

Parameter	F1	F2	F3	F4	A1	A2	A3
Age (years)	67.8±9.2	67.9±5.0	72.5±7.5	69.2±8.1	68.8±7.5	70.0±8.0	68.7±9.0
Sex distribution							
No. of men	2	9	15	49	24	50	1
No. of women	2	4	6	36	16	30	2
BMI (kg/m2)	22.0±1.9	22.7±2.8	24.0±2.5	23.0±3.1	23.5±3.7	23.0±2.6	22.5±1.9
HCV Serogroup*	(1)	(3)	(4)	(17)	(13)	(12)	(0)
No. of type 1	2	8	14	59	22	58	3
No. of type 2	1	2	3	9	5	10	0
HCV RNA titer (KIU/mL)*	1473.6±1706.3 (1)	884.5±807.0 (7)	1302.5±941.9 (6)	1279.7±1844.0 (23)	1731.5±2349.7(12)	1044.9±1151.9 (25)	884.9±168.9 (0)
ALT (U/L)	52.0±30.1	28.6±11.9	53.0±33.1	63.9±39.1	42.9±31.5	65.1±38.2	68.7±33.4
AST (U/L)	42.7±10.8	32.3±11.1	54.6±33.1	65.9±29.2	45.9±26.6	66.2±30.0	69.3±12.7
Glucose (mg/dL)	95.8±11.3	116.5±32.7	120.3±22.0	128.7±50.0	124.4±41.0	124.7±46.7	138.3±13.6
Total cholesterol (mg/dL)	160.8±27.3	182.5±36.8	161.2±37.0	154.7±112.7	164.1±38.4	157.9±115.7	119.3±16.7
Triglyceride (mg/ dL)	96.0±49.1	102.7±33.5	95.8±37.2	93.1±47.5	92.7±38.2	95.3±47.8	103.0±19.3
HDL (mg/dL) *	46.8±16.2	56.3±16.5	43.1±12.1 (1)	42.7±13.6 (2)	49.0±14.3 (1)	42.8±13.5 (2)	26.0±8.7
LDL (mg/dL) *	94.8±19.6	105.7±34.2	97.9±30.5 (1)	94.2±114.7 (2)	96.1±30.4 (1)	97.0x118.2 (2)	72.7±9.3

Data are the means± standard deviation. BMI body mass index (the individual’s body weight [in kilograms] divided by the square of his or her height [in meters]).

*Numbers in parentheses are the number of patients with missing data.

### Tissue classification by texture analysis

**Fig 2:**
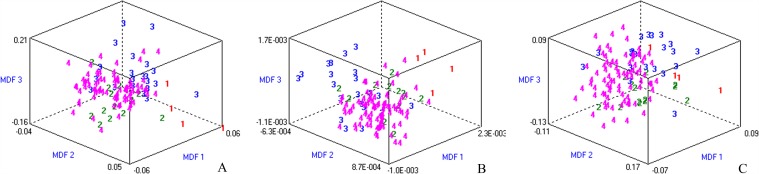
Discrimination of fibrosis stages based on all patient dataset. Misclassification rates were 28.46%, 35.77% and 20.33% in T2W **(A)**, T1W **(B)** and EOB-HP **(C)** images, respectively. The three-dimensional distribution of data vectors is based on the top three of the 30 texture features that were extracted using Fisher coefficients + classification error probability combined with average correlation coefficients + mutual information coefficients (Fisher+POE+ACC+MI) methods, followed by linear discrimination analysis /1-nearest neighbor (LDA/1-NN) classification: fibrosis stage 1 (1), stage 2 (2), stage 3 (3), and stage 4 (4). Most discriminating factor1 (MDF1), MDF 2 and MDF 3 are the most discriminating feature axes used in LDA to represent the classification graphically.

**Fig 3:**
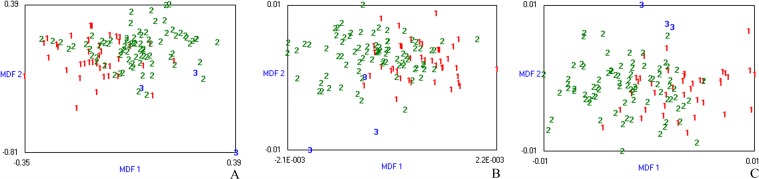
Discrimination of necroinflammatory activity grades based on all patient dataset. Misclassification rates were 34.15%, 25.20% and 28.46% in T2W **(A)**, T1W **(B)** and EOB-HP **(C)** images, respectively. The three-dimensional distribution of data vectors is based on the top three of the 30 texture features that were extracted using Fisher+POE+ACC+MI method, following by LDA/1-NN classification: necroinflammatory activity grade 1 (1), grade 2 (2) and grade 3 (3). MDF1 and MDF 2 are the most discriminating features axes used in LDA to represent the classification graphically.

30 texture features were selected with Fisher +POE+ACC+ MI methods in MaZda from 279 original parameters calculated in all patients for classifying the fibrosis stage or necroinflammatory activity grade in T2W, T1W and EOB-HP images, respectively. The misclassification rates, calculated across all images, were 28.46% for fibrosis staging and 34.15% for necroinflammatory activity grading using LDA in combination with 1-NN classification (LDA/1-NN) in T2W imaging depending on the method of feature selection (Fisher+POE+ACC+MI), 35.77% for fibrosis staging, 25.20% for necroinflammatory activity grading in T1W imaging, 20.33% for fibrosis staging and 28.46% for necroinflammatory activity grading in EOB-HP imaging, respectively. The clusters of samples were presented in two- or three-dimensional space of selected features, demonstrating the graphical relationship between the MDFs. The number of MDF axes is less than the number of the output classes by one, and so the sample distributions are visualized in three-dimensional space for classification of the fibrosis stages (F1~4) and in two-dimensional space for classification of necroinflammatory grades (A1~3) (Figs. 2 and 3).

**Fig 4:**
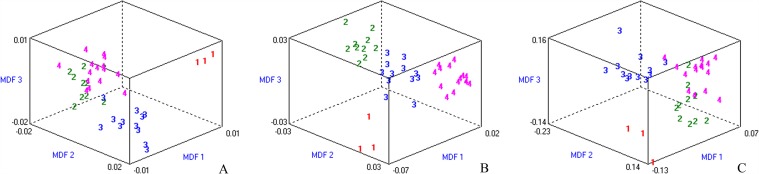
Discrimination of fibrosis stages based on patient group with necroinflammatory activity grade 1. Misclassification rates were 5.00%, 0% and 12.50% in T2W **(A)**, T1W **(B)** and EOB-HP **(C)** images, respectively. The three-dimensional distribution of data vectors is based on the top three of the 30 texture features that were extracted using Fisher+POE+ACC+MI method, followed by LDA/1-NN classification: fibrosis stage 1 (1), stage 2 (2), stage 3 (3), and stage 4 (4). MDF1, MDF 2 and MDF 3 are the most discriminating features axes used in LDA to represent the classification graphically.

**Fig 5:**
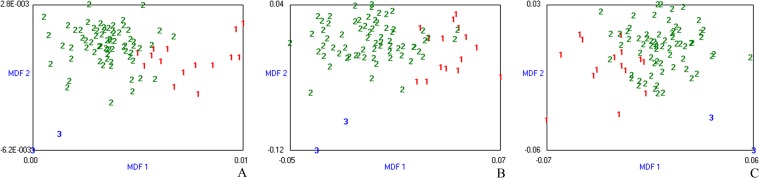
Discrimination of necroinflammatory activity grades based on patient group with fibrosis stage 4. Misclassification rates were 5.88%, 12.94% and 11.76% in T2W **(A)**, T1W **(B)** and EOB-HP **(C)** images, respectively. The three-dimensional distribution of data vectors is based on the top three of the 30 texture features that were extracted using Fisher +POE+ACC +MI method, following by LDA/1-NN classification: necroinflammatory activity grade 1 (1), grade 2 (2) and grade 3 (3). MDF1 and MDF 2 are the most discriminating features axes used in LDA to represent the classification graphically.

To avoid interference between fibrosis stage and necroinflammatory activity grade on texture features, the classification in the subgroups of necroinflammatory activity grades or fibrosis stages was further investigated. Only A1 group and F4 group met the condition that the sample size for each subgroup must exceed one. Further classification of A1 group for fibrosis staging and F4 group for necroinflammatory activity grading was run. 30 texture features were extracted using the same Fisher+POE+ACC+MI methods as before in MaZda in T2W, T1W and EOB-HP images, respectively. LDA/1-NN resulted in misclassification rates of 5.00% for fibrosis staging of A1 group and 5.88% for necroinflammatory activity grading of F4 group in T2W images. LDA/1-NN resulted in misclassification rates of 0% for fibrosis staging of A1 group and 12.94% for necroinflammatory activity grading of F4 group in T1W images. LDA/1-NN resulted in misclassification rates of 12.50% for fibrosis staging of A1 group and 11.76% for necroinflammatory activity grading of F4 group in EOB-HP images (Table 4). The separation of clusters reflects better discrimination in A1 or F4 groups for fibrosis staging or necroinflammatory activity grading than that in the all patient dataset (Figs. 4 and 5).

**Table 4:** Results of texture-based classification of liver fibrosis stages or necroinflammatory activity grades of chronic liver hepatitis C calculated for T2W, T1W and EOB-HP imaging sequences, according to linear discrimination analysis /1-nearest neighbor (LDA/1-NN) classification method.

	T2W (mis %)	T1W (mis %)	EOB-HP (mis %)
Classification of fibrosis staging in all patients	28.46	35.77	20.33
Classification of necroinflammatory activity grading in all patients	34.15	25.20	28.46
Classification of fibrosis staging in A1 patient group	5.00	0	12.50
Classification of necroinflammatory activity grading in F4 patient group	5.88	12.94	11.76

Misclassification percentage (mis %) was given in columns.

## Discussion

Our results demonstrated that texture features can not only differentiate normal liver from cirrhotic liver, but also have the ability to stratify the cirrhosis severity [13]. Furthermore, texture features showed the ability to evaluate the necroinflammatory activity grade as well as fibrosis stage, in contrast to many other methods that are more useful in discriminating the fibrosis stage rather than necroinflammatory activity grade. In the previous literature, the contrast enhancement index on EOB-HP showed a significant correlation with fibrosis stage but no correlation with necroinflammatory activity grades [11], and liver stiffness measurement using transient elastography could accurately reflect the amount of fibrosis but not determine the grade of necroinflammatory activity [29]. The discriminating ability of texture analysis revealed in this study is speculated to relate to the histopathological characteristics. The necroinflammatory process in chronic viral hepatitis, characterized by lymphocyte infiltration and hepatocyte damage [30, 31], is surmised to destroy the homogeneity of the liver parenchyma, which could be reflected by various texture features. The influence of fibrosis over texture could be deduced from the formation of fibrous septa and variously sized nodules [30, 31].

Until now, no widely accepted criterion for inclusion of texture features in evaluating MR images has been available. Past studies on texture analysis may have focused on all or some of the above parameters. In the study of Kato et al [32], the degree of fibrosis was accurately correlated with seven texture features. Given the diversity of derivation approaches of texture features, we expanded the data pool that included 279 texture parameters in this study, and 30 texture parameters were selected as independent variables, being relatively more numerous than those in the previous studies [29, 32–34]. In our opinion, a big data pool may be of benefit to flexible selection and application of texture parameters. Even though texture analysis was based on the same data pool, mostly different texture parameters were selected for different purposes, and these selected texture features could be used to discriminate either the fibrosis stage or necroinflammatory activity grade.

In this study, the misclassification rates for fibrosis staging in A1 group were much less than those for fibrosis staging in all patients in T2W, T1W or EOB-HP images. This suggests that the discriminating ability for classification of fibrosis stages can be improved when the influence of necroinflammatory activity grades is eliminated. Because progression of the fibrosis stage and necroinflammatory activity grade are not necessarily parallel in chronic hepatitis C, patients with the same fibrosis stage may have different necroinflammatory activity grades, and so the dataset containing different necroinflammatory activity grades will decrease the accuracy of classification of fibrosis stages using texture analysis method. Likewise, the accuracy of activity grade classification was also improved after the dataset was stratified in terms of the fibrosis stage. So we hypothesize that the texture features are related to both the fibrosis stage and necroinflammatory activity grade in chronic hepatitis C, and that furthermore, the influences on texture parameters from fibrosis stage and necroinflammatory activity grade may overlap to some extent. The subgroups divided by fibrosis or necroinflammatory activity degrees can be more accurately classified for activity grading or fibrosis staging based on the texture analysis method.

We also note that the differentiation ability for fibrosis staging is as good as that of necroinflammatory activity grading, which extends the application of texture analysis in the evaluation of chronic hepatitis C. This finding is a good proof for the powerful effect of texture analysis, which can be attributed to the enormous dataset calculated by many different methods including histogram, gradient, run-length matrix, co-occurrence matrix, autoregressive model and wavelet transformation methods. In this way the useful texture parameters selected differ according to the classification purpose and produce the optimum composition leading to the best results in either fibrosis or necroinflammatory activity classification.

There are several limitations in this study. First, we did not divide the patient population into a training and a test dataset, because of the small size of some subsets. We designed this study as a preliminary study that focused on the general feasibility of texture-based classification of fibrosis or necroinflammatory activity degrees, rather than constructing a working classification model at present. In addition, the 1-NN classifier using leave-one-out technique implemented in the MaZda program does not require a separate training dataset [35]. Second, the small sample size of patients with F1A2, F1A3, F2A3 and F3A3 was another drawback of this study, and so we only classified the A1 group for fibrosis staging, and F4 group for necroinflammatory activity grading, which was also limited by its retrospective nature.

In conclusion, based on the texture parameters calculated in T2W, T1W and EOB-HP images from patients with chronic hepatitis C, we conclude that texture analysis with the input dataset categorized according to activity or fibrosis degree shows promise as a useful quantitative tool for comprehensively evaluating the progress of fibrosis and necroinflammatory activity.

## Supporting Information

S1 Dataset Demographic variables: clinical data of 123 patients. (XLS)

S2 Dataset Texture features: texture parameters of 123 patients calculated by MaZda software for distinguishing fibrosis stages. (ZIP)

S3 Dataset Texture features: texture parameters of 123 patients calculated by MaZda software for distinguishing necroinflammatory activity grades. (ZIP)
